# Can a Strategic Pipeline Initiative Increase the Number of Women and Underrepresented Minorities in Orthopaedic Surgery?

**DOI:** 10.1007/s11999-016-4846-8

**Published:** 2016-04-25

**Authors:** Bonnie S. Mason, William Ross, Gezzer Ortega, Monique C. Chambers, Michael L. Parks

**Affiliations:** 1 Nth Dimensions Inc, 22 North Morgan Street, Suite 113, Chicago, IL 60607 USA; 2 Department of Surgery, Howard University Hospital, Washington, DC USA; 3 Department of Orthopaedic Surgery, Southern Illinois University, Springfield, IL USA; 4 Hospital for Special Surgery, New York, NY USA

## Abstract

**Background:**

Women and minorities remain underrepresented in orthopaedic surgery. In an attempt to increase the diversity of those entering the physician workforce, Nth Dimensions implemented a targeted pipeline curriculum that includes the Orthopaedic Summer Internship Program. The program exposes medical students to the specialty of orthopaedic surgery and equips students to be competitive applicants to orthopaedic surgery residency programs. The effect of this program on women and underrepresented minority applicants to orthopaedic residencies is highlighted in this article.

**Questions/purposes:**

(1) For women we asked: is completing the Orthopaedic Summer Internship Program associated with higher odds of applying to orthopaedic surgery residency? (2) For underrepresented minorities, is completing the Orthopaedic Summer Internship Program associated with higher odds of applying to orthopaedic residency?

**Methods:**

Between 2005 and 2012, 118 students completed the Nth Dimensions/American Academy of Orthopaedic Surgeons Orthopaedic Summer Internship Program. The summer internship consisted of an 8-week clinical and research program between the first and second years of medical school and included a series of musculoskeletal lectures, hands-on, practical workshops, presentation of a completed research project, ongoing mentoring, professional development, and counselling through each participant’s subsequent years of medical school. In correlation with available national application data, residency application data were obtained for those Orthopaedic Summer Internship Program participants who applied to the match between 2011 through 2014. For these 4 cohort years, we evaluated whether this program was associated with increased odds of applying to orthopaedic surgery residency compared with national controls. For the same four cohorts, we evaluated whether underrepresented minority students who completed the program had increased odds of applying to an orthopaedic surgery residency compared with national controls.

**Results:**

Fifty Orthopaedic Summer Internship scholars applied for an orthopaedic residency position. For women, completion of the Orthopaedic Summer Internship was associated with increased odds of applying to orthopaedic surgery residency (after summer internship: nine of 17 [35%]; national controls: 800 of 78,316 [1%]; odds ratio [OR], 51.3; 95% confidence interval [CI], 21.1–122.0; p < 0.001). Similarly, for underrepresented minorities, Orthopaedic Summer Internship completion was also associated with increased odds of orthopaedic applications from 2011 to 2014 (after Orthopaedic Summer Internship: 15 of 48 [31%]; non-Orthopaedic Summer Internship applicants nationally: 782 of 25,676 [3%]; OR, 14.5 [7.3–27.5]; p < 0.001).

**Conclusions:**

Completion of the Nth Dimensions Orthopaedic Summer Internship Program has a positive impact on increasing the odds of each student participant applying to an orthopaedic surgery residency program. This program may be a key factor in contributing to the pipeline of women and underrepresented minorities into orthopaedic surgery.

**Level of Evidence:**

Level III, therapeutic study.

## Introduction

Healthcare disparities have become a recent focus of literature in the United States [2, 4, 8, 9, 11, 12]. The preparation of a physician workforce that mirrors the gender, racial, and ethnic makeup of the population is an important step toward eliminating these disparities [7]. The current composition of the physician workforce in the United States has failed to reflect the changes seen in its population. One study states that the inability of the overall health profession to keep pace with the US population is a greater contributor to health disparities than access to care [5]. Regarding specialty fields of medicine, the lack of physician workforce diversity for all specialties is well documented in the literature [3, 10]. Orthopaedic surgery, in particular, is among the least diverse specialties for underrepresented minorities (eg, with only 3% of US orthopaedic surgeons of African American descent) and is the least diverse for women with females accounting for only 5% of all practicing orthopaedic surgeons while composing nearly 50% of the total population [3]. However, there are data to support the efficacy of certain strategies that increase physician workforce diversity. These include (1) early exposure to specialty fields; (2) addressing educational gaps; (3) mentoring; (4) the presence of and interaction with faculty reflective of women and underrepresented minority groups; and (5) the development of an institutional culture or network that is supportive of women and minority physicians [1, 6].

To address the low numbers of women and underrepresented minorities in orthopaedics, Nth Dimensions, a not-for-profit founded by orthopaedic surgeons in 2002 and formally incorporated in 2004, has developed and implemented a longitudinal pipeline curriculum consisting of 3 Phases (Table 1), which engages 300 to 400 US medical students annually from more than 40 medical schools. This article addresses the impact of Phase 2, the Nth Dimensions Orthopaedic Summer Internship, which specifically pairs selected first-year medical students with practicing surgeons for clinical shadowing and research and subsequent programming for the purpose of examining its effectiveness of this discrete intervention that only a limited number of students are awarded annually.

**Table 1:** The three sequential phases of the Nth Dimensions Pipeline Initiative Curriculum^TM^

Nth Dimensions Pipeline Initiative Curriculum^TM^	Title	Key program goals	Key program collaborations
Phase I	Clinical Correlations and Sawbones Bioskills Workshops	Early awareness and exposure	Local, volunteer orthopaedic surgeons and medical schools of historically black colleges and universities
Phase II	Orthopaedic Summer Internship Program	Specialty immersion	40 volunteer board-certified, orthopaedic surgeons from 32 US academic centers and private practices
Phase III	Ongoing Mentoring and Professional Development	Annual didactics on nonclinical skills-building, board preparation, annual exposure to underrepresented minorities and women orthopaedic surgeons	Orthopaedic associations, eg, American Academy of Orthopaedic Surgeons, J. Robert Gladden Orthopaedic Society

With multiple participants of the Nth Dimensions’ Orthopaedic Summer Internship immersion program now entering clinical practice, the effectiveness of the program warrants exploration. In particular, the program’s focus on women and underrepresented racial/ethnic minorities should translate into measurably more individuals applying to orthopaedic surgery residency, which ultimately could lead to more diversity in the orthopaedic physician workforce. In addition to thorough evaluation of the overall effectiveness of this program in increasing currently underrepresented applicants to orthopaedic surgery residencies, an evaluation of acceptance rates into these same programs also warrants future consideration.

We therefore asked: (1) Is completion of the Nth Dimensions Orthopaedic Summer Internship and additional programming associated with higher odds of women applying to orthopaedic surgery residency? (2) Is completion of the Nth Dimensions Orthopaedic Summer Internship and additional programming associated with higher odds of underrepresented minorities applying to orthopaedic residency?

## Materials and Methods

This is a retrospective observational cohort study of 118 medical students from 29 accredited US medical schools, who were awarded a position in the Nth Dimensions/American Academy of Orthopaedic Surgeons (AAOS) Orthopaedic Summer Internship Program. Between 2005 and 2012, eight cohorts of first-year medical students were awarded an internship during the 8-week Orthopaedic Summer Internship Program at one of 40 US medical schools and community orthopaedic practices. In addition, students participated in annual educational programming, skills-building, and ongoing mentoring throughout the remainder of medical school and were observed through the match process as fourth-year medical students. Their decision to apply to a self-elected specialty was followed to ascertain both application (retention) and match rates in orthopaedic surgery and other specialties.

In total, 118 US medical students, in good academic standing at an accredited US allopathic or osteopathic medical school, completed the Nth Dimensions/AAOS’ Orthopaedic Summer Internship Program during the summer bridging their first and second years in medical school. These applicants were made aware of the program by several methods including: exposure to Nth Dimensions programs during our hands-on workshops, recommendations from current and former preceptors as well as past participants, referrals from academic deans already aware of the program, and materials on the Nth Dimensions website.

The inclusion criterion for this report is all students who successfully completed the program (n = 118). Exclusion criterion for the first arm of this study was students who did not complete the entire program (n = 2). Among the 118 scholars, the eight cohorts were composed of 48 (41%) women; the cohorts were ethnically composed of 82 (69%) black, 16 (14%) Latino, 11 (9%) white, six (5%) Native Americans/Indian, and three (3%) Asian participants. Fifty Orthopaedic Summer Internship scholars applied for an orthopaedic residency position. The overall match rate across our eight cohorts was 76%; this data has been summarized for each cohort (Table 2). Data regarding students who applied and/or matched into non-orthopaedic specialties were collected and reported in the additional findings of this particular study. Six participants were lost to follow-up, because their specialty and residency location remain unknown. This yields 112 Orthopaedic Summer Internship scholars whose matriculation after the match was known and reported in this study.

**Table 2:** Overall orthopaedic surgery application and match rates for all Orthopaedic Summer Internship scholars

Cohort (program year)	Match year	Participants	Orthopaedic applicants	Total matched	Overall match rate	Applied match rate
I (2005)	2008	10	4	3	30%	75%
II (2006)	2009	17	8	6	35%	75%
III (2007)	2010	14	6	2	14%	33%
IV (2008)	2011	15	5	3	20%	60%
V (2009)	2012	7	3	3	43%	100%
VI (2010)	2013	17	5	5	29%	100%
VII (2011)	2014	20	6	4	20%	67%
VIII (2012)	2015	18	13	12	67%	92%
	Total	118	50	38	32%	76%

### Nth Dimensions Pipeline Curriculum Description

This 4-year developmental program (as detailed subsequently) is designed to expose medical students nationwide to the field of orthopaedics early in their medical education. After initial exposure to orthopaedic surgery through hands-on, “sawbones” workshops, the first phase of Nth Dimensions programming; qualified students are selected for the second phase, the Orthopaedic Summer Internship Program. By a stringent application process paralleling that of medical school. Qualified students are those who are in their first year of medical school and who are in good standing with their respective academic institutions. Orthopaedic Summer Internship applicants were required to submit an application, personal statement, two letters of recommendation, documentation of good academic standing in medical school, and subjected to a panel-led interview. The panel, composed of surgeons from the AAOS, Nth Dimensions, and prior Orthopaedic Summer Internship scholars, interviewed each student and provided an assessment on the applicant’s desire to pursue a career in orthopaedics, mentorship opportunities, letters of recommendation, and feedback from the interview panel. A Likert scale evaluation is used based on the aforementioned applicant criteria. Currently, there is a 3 to 1 ratio of applicants for the available internship positions. The more than 40 orthopaedic surgeon preceptors, from a variety of academic institutions and community practices, are members of varying ethnic and gender minority groups who have also been specifically selected because of their teaching and mentoring achievements in the field of orthopaedics. This 4-year developmental program (as detailed subsequently) is designed to expose medical students to the field of orthopaedics early in their educational process. The students receive core mentorship from their internship preceptor throughout medical school and are encouraged to develop mentoring relationships with additional surgeons and residents through scheduled interactions and mentoring activities during annual programs. Continued exposure through clinical skills didactics, professional development workshops, and ongoing mentorship helps support and develop students into competitive applicants in conjunction with exposure to the J. Robert Gladden Orthopaedic Society and the AAOS.

To assess the effectiveness of the Nth Dimensions Orthopaedic Summer Internship and additional programming, the primary outcome is application to orthopaedic surgery residency. We analyzed our data focused on women and underrepresented minorities, the latter subdivided into black and Latino students. We then evaluated the proportions of women and underrepresented minority students who completed the Orthopaedic Summer Internship and then applied to an orthopaedic residency program and compared them with same-year national proportions who applied to orthopaedic programs. We also identified other nonorthopaedic training programs to which the Orthopaedic Summer Internship scholars applied.

### Statistical Analysis

Descriptive statistics focused on frequencies and proportions were obtained for retention and match acceptance for all eight cohorts of the Orthopaedic Summer Internship participants. The proportion of both Orthopaedic Summer Internship and non-Orthopaedic Summer Internship orthopaedic surgery applicants for four cohorts was based on the national data that is available (2011–2014). Odds ratios (ORs) with 95% confidence intervals (CIs) were calculated to test the association of program completion and application to an orthopaedic surgery residency stratified by gender (women, men) and race/ethnicity (underrepresented minorities [blacks and Latinos] versus nonunderrepresented minorities). All statistical analyses were performed using Stata 13.1 (College Station, TX, USA) with significance level set at p < 0.05. An institutional review board waiver was obtained before conducting this study (institutional review board #11053/eCAP# 2015-140).

## Results

### Women Applicants to Orthopaedic Residencies

For women, Orthopaedic Summer Internship completion was associated with increased odds of applying to orthopaedic surgery residency from 2011 to 2014 (after Orthopaedic Summer Internship: nine of 29 [31%]; national controls, excluding Orthopaedic Summer Internship applicants: 800 of 77,514 [1%]; OR, 43.2; 95% CI, 17.2–99.6; p < 0.001; Table 3). When stratified by year, there were two of seven (29%) women who completed Orthopaedic Summer Internship who applied to orthopaedic surgery in 2011 compared with 224 of 18,953 (1%) non-Orthopaedic Summer Internship applicants nationally (OR, 33.4; 3.2–205.3; p = 0.003); in 2012, two of five (40%) compared with 187 of 19,072 (1%) (OR, 67.3; 5.6–590; p = 0.001); in 2013, two of seven (29%) compared with 158 of 19,662 (1%) (OR, 49.4; 4.7–303.8; p = 0.001); and in 2014, three of 10 (30%) compared with 231 of 19,827 (1%) (OR, 36.6; 6–160.3; p < 0.001).

**Table 3:** Women applicants to orthopaedics, OSI versus national controls

Year	Women who went through OSI (n = 29)	Women from national controls (n = 77,514)	Odds ratio (95% CI)	p value
2011	2/7 (29%)	224/18,953 (1%)	33.4 (3.2–205.3)	0.003
2012	2/5 (40%)	187/19,072 (1%)	67.3 (5.6–590)	0.001
2013	2/7 (29%)	158/19,662 (1%)	49.4 (4.7–303.8)	0.001
2014	3/10 (30%)	231/19,827 (1%)	36.6 (6–160.3)	< 0.001
Overall	9/29 (31%)	800/77,514 (1%)	43.2 (17.2–99.6)	< 0.001

OSI = Orthopaedic Summer Internship; CI = confidence interval.

### Underrepresented Minority Applicants to Orthopaedic Residencies

For underrepresented minorities, Orthopaedic Summer Internship completion was also associated with increased odds of orthopaedic applications from 2011 to 2014 (after Orthopaedic Summer Internship: 15 of 48 [31%]; non-Orthopaedic Summer Internship applicants nationally: 782 of 25,676 [3%]; OR, 14.5; 7.3–27.5; p < 0.001; Table 4). Stratified analyses revealed that in 2011, there were five of 13 (38%) underrepresented minorities who completed Orthopaedic Summer Internship that chose orthopaedic surgery compared with 202 of 6138 (3%) nationally (OR, 18.4; 4.7–64.2; p < 0.001); in 2012, there were three of seven (43%) compared with 189 of 6175 (3%) (OR, 23.8; 3.4–141.2; p < 0.001; p < 0.001); in 2013, there were five of 16 (31%) compared with 162 of 6552 (2%) (OR, 17.9; 4.8–56.6; p < 0.001); and in 2014, there were two of 12 (17%) compared with 229 of 6811 (OR, 7.2; 0.74–36.3; p = 0.043).

**Table 4:** Underrepresented minority applicants to orthopaedics, OSI versus national controls

Year	OSI (n = 48)	National controls (n = 25,676)	Odds ratio (95% CI)	p value
2011	5/13 (38%)	202/6138 (3%)	18.4 (4.7–64.2)	< 0.001
2012	3/7 (43%)	189/61,752 (3%)	23.8 (3.4–141.2)	< 0.001
2013	5/16 (31%)	162/6552 (2%)	17.9 (4.8–56.6)	< 0.001
2014	2/12 (17%)	229/6811 (3%)	7.2 (0.74–36.3)	0.043
Overall	15/48 (31%)	782/25,676 (3%)	14.5 (7.3–27.5)	< 0.001

OSI = Orthopaedic Summer Internship; CI = confidence interval.

When stratified by race, we found similar results. For Black students applying from 2011 to 2014, Orthopaedic Summer Internship completion was associated with increased applications to orthopaedics (after Orthopaedic Summer Internship: 12 of 42 [29%]; non-Orthopaedic Summer Internship applicants nationally: 397 of 12,519 [3%]; OR, 15.9; 7.9–30.7; p < 0.001). For Latino students over the same years, Orthopaedic Summer Internship completion was also associated with increased applications to orthopaedics (after Orthopaedic Summer Internship: three of six [50%]; non-Orthopaedic Summer Internship applicants nationally: 385 of 12,372 [3%]; OR, 32.1; 4.3–240.4; p < 0.001).

### Specialties Chosen Other Than Orthopaedics

**Fig. 1:**
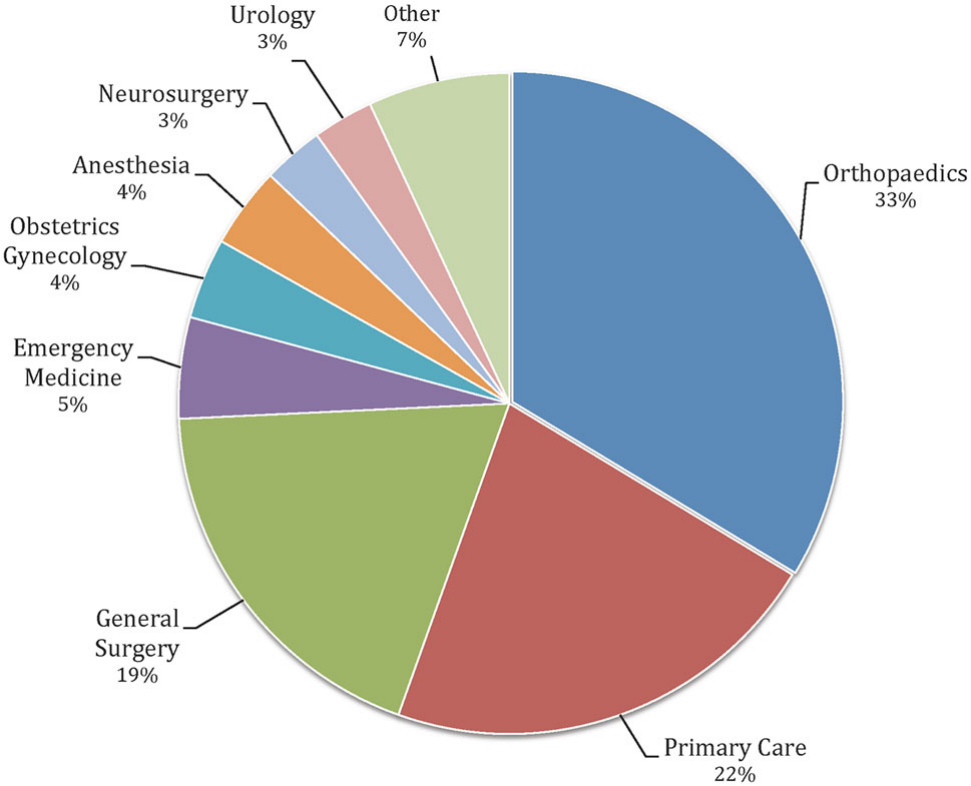
Orthopaedic Summer Internship match rates by specialty are shown. Other includes ophthalmology (2%), radiology, diagnostic (2%), physical medicine and rehabilitation (1%), transitional year (1%), and preventive medicine (1%). Primary care includes family medicine/internal medicine/pediatrics.

Overall, for those who completed the Orthopaedic Summer Internship but did not apply to orthopaedics from 2011 to 2014 (n = 40), specialty choices were: primary care (22%), general surgery (19%), emergency medicine (5%), obstetrics-gynecology (4%), anesthesia (4%), neurosurgery (3%), urology (3%), and other (7%) (Fig. 1).

## Discussion

In response to the lack of diversity among the US orthopaedic physician workforce, Nth Dimensions initiated a longitudinal, developmental program that includes the Orthopaedic Summer Internship Program designed to increase the number of women and underrepresented minorities in the orthopaedic specialty. Current evidence supports that existing healthcare disparities may be diminished when the physician workforce more directly reflects the diversity in the US population [5]. Although the long-term effects of this program remain to be seen, effectiveness in the short term was assessed based on participating students’ application to orthopaedic surgery residency programs. Therefore, for women and underrepresented minorities, we evaluated the program’s success in increasing the proportion of these students applying to these programs. The overall goal of this evaluation is to demonstrate the effectiveness of the program with the intent to expand its reach nationally and to encourage similar programs in other medical specialties where women and minorities are underrepresented.

There were several limitations to this study. First, national comparison data detailing the application rates of medical students based on gender, race, and ethnicity have not been released from governing associations for all of the years corresponding to our respective cohorts. Therefore, only half of our cohorts were able to be fully evaluated in comparison to national averages. In addition, complete national data regarding acceptance or “match” rates into orthopaedic surgery training programs of medical students based on gender, race, and ethnicity are currently publically unavailable. The difficulty in obtaining this information highlights the need for greater transparency of this data. We are, therefore, unable to compare the match rates of those women and minority students who participated in the Orthopaedic Summer Internship versus those who did not. When compared with the available data, our overall application rates to competitive residency programs (Tables 3, 4) were above national averages for females and underrepresented minorities, as indicated by recently released data from the 2014 Association of American Medical Colleges’ (AAMC) Electronic Residency Application Service (ERAS). From 2011 to 2014, there were a total of 800 female applicants, nationally, to orthopaedic residency programs out of 77,514 total female applicants to all residency programs (1%), compared with 9 female orthopaedic applicants out of 29 total female applicants (31%) from our four cohorts. Second, our current data end with acceptance into a residency program. Additional followup, that assesses completion of residency, will be a valuable determinant of the long-term success of the program. Preliminary evaluation of post-match data reveals that all (100%) students recruited and retained through the Nth Dimensions Orthopaedic Summer Internship and additional programming have completed or remain in the orthopaedic residency in which they were matched.

Additional study limitations concern the potential for self-selection bias among participants. Although many of our Orthopaedic Summer Internship participants are introduced to orthopaedic surgery through our hands-on workshops in the first phase of our additional programming, some participants may already have exposure and interest in orthopaedics. Although our study clearly demonstrates that the students who participate in the Orthopaedic Summer Internship are more likely to apply to orthopaedic residency programs, it is difficult to determine to what degree some of these students were influenced by preexisting interest in addition to their contact with the program.

An additional limitation is the relatively small numbers in our cohorts in comparison to data encompassing all applicants to orthopaedic residency programs during the years evaluated. In addition, six students who completed the Orthopaedic Summer Internship were lost to followup, so it is possible that our reported outcomes may underestimate (or possibly overestimate) the effectiveness of our program. When compared with all orthopaedic applicants, an additional 50 applicants from our program might not appear significant. However, our comparison is not to all applicants but is stratified for comparison to women, Latino, and Black applicants specifically.

When compared with the significantly smaller numbers of women and underrepresented minority applicants, our numbers take on a much greater statistical significance and therefore a greater contribution to the diversity of the orthopaedic physician workforce.

Finally, cost is also a potential limitation of the program. Quantifying an accurate cost-benefit ratio remains a challenge at best. Currently, we have three applicants for every one available position. The demand for the program highlights our support for its expansion. As the program grows to accommodate more participants, then the cohort grows significantly with greater power to evaluate in comparison to national controls. A cost-efficiency analysis is a goal of future studies in an effort to optimize the resources available and maximize the impact of this program in diversifying the physician workforce.

This study suggests that the Nth Dimensions Orthopaedic Summer Internship and additional programming is effective in significantly increasing the odds of its participants (women and underrepresented minorities) applying to an orthopaedic residency program. It is important to note that though this study was not designed to show a cause-effect relationship between participation in the Orthopaedic Summer Internship and subsequent application to an orthopaedic residency program, a strong association between the two is demonstrated. The positive impact that this pipeline program demonstrates lends support to efforts promoting the ongoing exposure, preparation, and mentorship of women and underrepresented minority applicants to orthopaedic programs. It is our hope that this program in particular and others such as the Perry Initiative, a national program originally designed to expose female high school students to orthopaedics, continue to garner support for expansion to serve greater numbers of interested medical students and perhaps be implemented in other fields of medicine and health sciences. To our knowledge, there is no published data regarding similar medical-school focused pipeline programs.

Medical schools and residency programs that have encountered Orthopaedic Summer Internship scholars are increasingly referring more students to the program. In addition, partnerships with private orthopaedic enterprises as well as medical schools are being explored and implemented to provide additional financial support to increase the number of program participants. Hopefully, the success of this program will stimulate further support of this segment of the physician diversity pipeline.
